# Trimethylamine *N*-Oxide: A Link among Diet, Gut Microbiota, Gene Regulation of Liver and Intestine Cholesterol Homeostasis and HDL Function

**DOI:** 10.3390/ijms19103228

**Published:** 2018-10-19

**Authors:** Marina Canyelles, Mireia Tondo, Lídia Cedó, Marta Farràs, Joan Carles Escolà-Gil, Francisco Blanco-Vaca

**Affiliations:** 1 Hospital de la Santa Creu i Sant Pau, Servei de Bioquímica-Institut d’Investigacions Biomèdiques (IIB) Sant Pau, 08041 Barcelona, Spain; mcanyelles@santpau.cat (M.C.); fblancova@santpau.cat (F.B.-V.); 2 Institut de Recerca de l’Hospital Santa Creu i Sant Pau-Institut d’Investigacions Biomèdiques (IIB) Sant Pau, 08025 Barcelona, Spain; lcedo@santpau.cat (L.C.); mfarras@santpau.cat (M.F.); 3 CIBER de Diabetes y Enfermedades Metabólicas Asociadas (CIBERDEM), 08907 Barcelona, Spain; 4 CIBER de Fisiopatología de la Obesidad y Nutrición (CIBEROBN), ISCIII, 08003 Barcelona, Spain; 5 Departament de Bioquímica, Biologia Molecular i Biomedicina, Universitat Autònoma de Barcelona, 08193 Barcelona, Spain

**Keywords:** trimethylamine, trimethylamine-*N*-oxide, intestinal microbiota, FMO3, reverse cholesterol transport, cholesterol homeostasis, atherosclerosis and cardiovascular disease

## Abstract

Recent evidence, including massive gene-expression analysis and a wide-variety of other multi-omics approaches, demonstrates an interplay between gut microbiota and the regulation of plasma lipids. Gut microbial metabolism of choline and l-carnitine results in the formation of trimethylamine (TMA) and concomitant conversion into trimethylamine-*N*-oxide (TMAO) by liver flavin monooxygenase 3 (FMO3). The plasma level of TMAO is determined by the genetic variation, diet and composition of gut microbiota. Multiple studies have demonstrated an association between TMAO plasma levels and the risk of atherothrombotic cardiovascular disease (CVD). We aimed to review the molecular pathways by which TMAO production and FMO3 exert their proatherogenic effects. TMAO may promote foam cell formation by upregulating macrophage scavenger receptors, deregulating enterohepatic cholesterol and bile acid metabolism and impairing macrophage reverse cholesterol transport (RCT). Furthermore, FMO3 may promote dyslipidemia by regulating multiple genes involved in hepatic lipogenesis and gluconeogenesis. FMO3 also impairs multiple aspects of cholesterol homeostasis, including transintestinal cholesterol export and macrophage-specific RCT. At least part of these FMO3-mediated effects on lipid metabolism and atherogenesis seem to be independent of the TMA/TMAO formation. Overall, these findings have the potential to open a new era for the therapeutic manipulation of the gut microbiota to improve CVD risk.

## 1. Introduction

Cardiovascular disease (CVD) accounts for approximately 17 million deaths worldwide each year and remains the main cause of mortality in the United States [1]. An impaired ability to eliminate cholesterol through bile acid excretion may be a risk factor for CVD development [2]. Environmental factors also play a major role in the progression of atherosclerosis and CVD [3]. Every individual has a large number of microorganisms shaping the gut microbiota, which are an intensively studied community of bacterial species [4]. The human gut microbiota represents more than 100 trillion microbes and 5000 different species, containing together around 5 million genes [5]. The combined genomes of the microbiota contain over 100-fold more unique genes than those encoded in the human genome [6], and these microbiota genes contribute significantly to our physiology and metabolism [7]. In recent years, the concept of pathological variation in the gut microbiota as the cause of several disease states has taken greater importance thanks to data from various rodent studies suggesting that dysbiosis contributes to the pathogenesis of diseases [8,9]. In that perspective, there is significant evidence supporting a role of the gut microbiota in cardiovascular health [10] and the onset and development of complex cardiometabolic diseases such as obesity, diabetes and metabolic syndrome [11,12]. Furthermore, the gut microbiota plays a critical role in plasma lipid levels, which was mainly found in plasma triglycerides and high-density lipoprotein (HDL) cholesterol levels [13]. Based on these studies, a link between microbiota, cardiometabolic diseases and CVD can be devised.

Several studies have identified novel microbial and mammalian cometabolic pathways where the microbiota could promote CVD pathogenesis via the formation of trimethylamine-*N*-oxide (TMAO) from trimethylamine (TMA) by host’s flavin monooxygenase 3 (FMO3) [14,15]. These studies reported that TMAO directly promoted atherosclerosis in mice. Additionally, as shown by two large prospective studies’ analyses in humans, plasma TMAO levels predicted CVD risk [14,15]. However, the underlying pathways whereby TMAO promotes atherosclerosis require further investigation and validation.

Methods to measure TMA and TMAO in plasma and urine include liquid chromatography-mass spectrometry, proton nuclear magnetic resonance spectrometry, headspace gas chromatography, electrospray ionization tandem mass spectrometry and matrix-assisted laser desorption/ionization time-of-flight mass spectrometry [16]. Concerning the reported reference values, the largest study performed with a healthy American population included 349 individuals and described values around 3.45 μmol/L [17]. Other studies that investigated healthy European individuals, reported values around 2.5 µmol/L in a cohort of 271 individuals [18] and values around 3.7 µmol/L in a cohort of 100 individuals [19].

In the present review, we aimed to describe and update the potential mechanisms by which TMAO production and FMO3 alter enterohepatic cholesterol and bile acid metabolism and, in consequence, impair a major HDL-mediated cardioprotective function.

## 2. TMA and TMAO

### 2.1. Metabolism of TMA and TMAO

TMAO was first described as an osmolyte of marine organism tissue [20]. TMAO is a small organic compound classified as an amine oxide and is formed in the liver from TMA. As a part of the microbial-mammalian cometabolism, TMA is generated by the action of gut microbiota using dietary precursors such as choline, choline-containing compounds, betaine or l-carnitine [16]. TMA is then converted into TMAO by FMO expressed in the liver. The intermediate γ-butyrobetaine (γBB) is formed in the conversion of l-carnitine to TMAO [21]. TMAO is then either transported to the tissues for accumulation as an osmolyte compound or, more commonly, cleared by the kidney from where TMAO is then excreted unchanged through the urine.

Among the five members of the FMO family, only FMO1 and FMO3 have the ability to oxidize TMA to TMAO—where FMO3 is the main isoform expressed in the human liver [22]. For FMO3, certain rare deleterious mutations in its gene are known to cause a reduced or absent TMAO formation, which in turn causes an accumulation of TMA. This autosomal recessive condition is called trimethylaminuria or “fish malodor syndrome” (OMIM 602079). Early reports of this condition date back to 1970 [23] and describe that individuals suffering from this disease experienced urine, sweat and breath that smelled like rotting fish.

In addition to its expression in the liver, FMO3 is also expressed in the lungs, adrenals and aorta. Interestingly, a sexually dimorphic expression pattern has been observed both in mice and humans, with females showing higher expression than males [24,25]. This gender difference may be explained by hormonal regulation: Testosterone is responsible for the lower hepatic FMO3 expression in males and estrogen induces FMO3 expression in females [24]. However, human studies have produced divergent results on gender-related differences in plasma TMAO concentrations. For instance, several studies investigating TMAO expression in humans did not find significant sex differences in plasma TMAO levels [18,19,26], whereas others reported higher ones either in females [22,27] or in males [28,29]. In a recent study involving a cohort of 648 individuals, males were found to have significantly higher levels of TMAO, even after adjusting for confounding factors such as age and kidney function [30]. Various variables including age, body mass index and blood pressure have been proposed as a cause of these conflicting findings since they were associated positively with the levels of TMAO [18,19,26]. More studies are required to clarify this conundrum.

Fasting plasma concentrations of TMAO exhibit a wide inter- and intra-individual variation [19]. The levels of circulating TMAO are affected by several factors, which include kidney function, diet, protein transport and, as stated before, the gut microbiota. Patients with chronic kidney disease (CKD) who are on haemodialysis show about 40-fold elevated TMAO levels compared with normal controls [28,31]. However, studies linking TMAO with the risk of cardiovascular events and mortality in CKD patients rendered inconclusive results. Some authors suggest that the higher TMAO levels may be confounded with impaired kidney function and poor metabolic control rather than history, presence or incidence of symptoms or events of coronary heart disease [28]. On the other hand, one study suggests that elevated TMAO levels are strongly associated with degree of renal function in CKD and that TMAO levels normalize after renal transplantation [32]. Additionally, TMAO levels correlate with increased systemic inflammation and it is an independent predictor of mortality in CKD patients [33]. These studies also suggest that TMAO may represent a new potentially modifiable CV risk factor for CKD patients [32,33]. A recent study showed that TMAO is a prognostic biomarker of kidney function in individuals with low renal function. The authors found that the metabolites derived from the gut microbiota strongly correlated with TMAO and that the magnitude of the correlation varied with kidney function independent of age, sex and baseline glomerular filtration rate [30].

Another important cause of plasma TMAO variability is diet. Food containing TMAO or its precursors increases blood and urine TMAO levels. Red meat, eggs and dairy products are all rich in TMA precursors, and therefore, a potential source of TMAO [34]. In addition, TMA and TMAO can also be acquired directly from fish and other seafood [34]. Overall, fish seems to have the highest source of TMAO, and post-ingestion studies show a marked increase in TMAO and related metabolites when compared with other foods enriched in carnitine or choline [34]. However, inconsistencies in findings remain since the results of several long-term studies did not indicate a strong effect of diet on TMAO plasma concentrations [18,19,26]. The organic cation transporter 2 (OCT2) located on the basolateral membrane of renal tubule cells is the key uptake transporter for TMAO and it may also be a major determinant of its variability [35].

Concerning the gut microbiota, several families of bacteria from the Firmicutes and Proteobacteria phyla isolated from commensal bacteria in the human intestine have been identified as choline and carnitine consumers and, therefore, potential TMA producers [36,37]. Pathways of TMA synthesis in the intestine have been described with a specific glycyl radical enzyme (GRE) including the GRE choline TMA-lyase (cutC) and its activator GRE activase (cutD), which uses choline as a substrate [38] and also, a two-component Rieske-type oxygenase/reductase (cntA/B), which uses carnitine and its γBB derivate as a substrate [39]. In a recent study of 648 individuals following a health coaching, determinants of gut microbiota and TMAO metabolism were identified [30]. The impact of diet (enriched in either meat or vegetal foods) on both the gut microbiota’s composition and TMAO was confirmed; the levels of TMAO were more affected by microbiota activity in individuals with higher kidney function. However, TMAO levels were significantly affected by the lack of TMAO filtering in individuals with low kidney function [30].

### 2.2. Genetic Determinants of TMAO

A great effort to find a relationship between gene expression and plasma TMAO levels was exerted over the last years. Various studies have focused on TMAO and CVD to determine the genetics that possibly underlie their relationship. Functional differences in FMO3 activity can occur in humans, secondary to variations within the *FMO3* gene. In addition to mutations causing trimethylaminuria, single nucleotide polymorphisms such as E158K and E308G have been reported to reduce FMO3 activity [40]. However, the implications of these polymorphisms on disease risk are complex and largely unknown due to inconclusive results [41,42,43].

Apart from *FMO3*, genome-wide association studies (GWAS) in male mice have allowed the identification of other genes related to TMAO metabolism. For instance, the *Slc30a7* gene has been found to be related to TMAO metabolism and is associated with a transporter of dietary zinc absorption (ZNT7) located on chromosome 3 [44]. Nonetheless, no significant threshold was observed in the human GWAS for the same gene [45].

A role for genetic methylation in TMAO homeostasis has recently been proposed by a study that reported an inversed association between plasma TMAO and the methylation capacity in humans [46]. However, an epigenome-wide study analyzed the methylation levels of CpG islands included in 463,995 loci finding no significant associations between methylation and circulating TMAO levels [47].

### 2.3. TMAO as a Plausible Contributor to Cardiovascular, Peripheral and Cerebrovascular Diseases

TMAO came recently under the spotlight due to its reported association with human CVD. In 2011, metabolomic studies identified three candidate molecules that were significantly correlated—after adjusting for traditional cardiac risk factors and medication usage—with CVD, which included the fasting TMAO, choline and phosphatidylcholine plasma levels [14]. Several years later, an association between carnitine concentration and the risk of coronary artery disease, peripheral artery disease and overall CVD was found to be affected by the microbiota metabolite TMAO [48]. The study concluded that carnitine was also an independent predictor of major adverse cardiovascular events (MACE), with TMAO as the main driver behind the association of cardiovascular risk [48]. Nonetheless, consumption of fish, which is high in TMAO, has long been associated with reduced CVD risk [49]. Other clinical trials also indicated that diets enriched in carnitine are associated with beneficial effects on CVD [50].

To better understand TMAO’s role in CVD, large population studies were performed with subjects undergoing elective diagnostic coronary angiography [15,51]. A direct relationship between increased plasma TMAO levels and the increased risk of MACE was observed. Furthermore, the inclusion of TMAO resulted in an improvement of risk estimation obtained by the traditional risk factors [15]. More recently, TMAO has been related to acute coronary syndromes in two different cohorts that showed an association of high TMAO plasma levels with an increased risk of MACE and mortality [52]. Additionally, high TMAO levels were also associated with a poor prognosis since they enhanced MACE risk and reinfarction in patients that had had acute myocardial infarction [52]. Finally, TMAO was also indicated as a secondary risk stratification biomarker of acute myocardial infarction to detect low-risk patients among the high-risk group [53].

Studies that focus on specific pathologies (e.g., heart failure) have also demonstrated a higher concentration of plasma TMAO compared to their respective controls. Furthermore, this higher concentration was associated with poor prognosis for heart failure patients and, consistently, plasma TMAO levels correlated positively with other cardiac biomarkers such as B-type natriuretic peptide [54].

An increase in the all-cause mortality and an improvement in risk estimation were also observed in patients with peripheral artery disease in relation to their TMAO plasma levels [55]. Increased choline and betaine concentrations also correlated with a higher risk of MACE and CVD. However, this correlation was only significant when higher plasma levels of TMAO were observed [51].

It should be noted that not all studies have demonstrated an association between TMAO and vascular diseases. For instance, a recent study reported a reduction in TMAO plasma levels and dysbiosis of the gut microbiota in patients that had a stroke or transient ischaemic attack [56]. The authors suggested that either the stroke event or the treatment may reduce TMAO and that the associated dysbiosis of the gut microbiota could be related with the differential role of TMAO in these pathologies [56]. Finally, other intermediate metabolites have been proposed to be involved in CVD. In patients with carotid atherosclerotic plaques, γBB and its precursor trimethyllysine were found to be associated with cardiovascular mortality [57].

The underlying mechanisms whereby TMAO contributes to CVD are not fully understood. The first hypothesis that explained the atherosclerotic role of TMAO stated a decrease in HDL levels. This was based on the inverse association between TMAO and HDL, which had been found in mice concomitant to a reduction in HDL-mediated reverse cholesterol transport (RCT, see Section 4 for more details) [14]. Nonetheless, human studies resulted in conflicting results concerning this topic as some authors reported significant lower HDL levels in high TMAO-expressing individuals with CVD [54,55], whereas others found a positive correlation between them [18]. Interestingly, a recent study reported no significant correlations between TMAO and the previously established blood markers for CVD, including the total cholesterol, low-density lipoprotein (LDL), HDL and triglycerides [30]. This might in part explain why TMAO has been found to be a prognostic marker for CVD beyond the traditional risk factors as previously indicated [52].

A recent study showed that inhibition of TMA lyases—enzymes expressed by gut microbes that convert choline to TMA—reduced atherosclerosis in mice [58]. TMAO-mediated atherosclerosis most likely occurs through multiple pathways. TMAO enhances the forward macrophage cholesterol transport via the upregulation of receptor CD36 (cluster of differentiation 36) and scavenger receptor A [14]. TMAO is also known to alter the enterohepatic cholesterol and bile acid metabolism, thereby impairing a major pathway required to eliminate cholesterol from the body, i.e., HDL-mediated RCT (see Section 4 for details) [48,59]. Other alternative mechanisms in relation to TMAO-mediated atherosclerosis include angiotensin II, which causes a prolongation of hypertension [60], the activation of nuclear factor κB signaling, which promotes vascular inflammation [61], and an enhanced platelet activation, which may promote a thrombosis effect [62].

Nonetheless, it should be noted that the findings of two recent experimental studies have cast doubts on the TMAO hypothesis [63,64]. In the first study, high doses of carnitine resulted in a significant increase in plasma TMAO levels in mice but, surprisingly, they inversely correlated with aortic atherosclerotic lesions [63]. The second study found that choline supplementation increased plasma TMAO in conventionally raised mice but not in germ-free mice. However, this treatment did not affect the atherosclerosis susceptibility [64].

## 3. HDL Function and CVD

The concept that HDL cholesterol protects against CVD was originally based on epidemiological data showing that low HDL cholesterol levels have a predictive factor of major adverse CVD events [65]. However, the inverse relationship between CVD risk and HDL is not strictly related to the amount of cholesterol transported by HDL, and the failure of various HDL-targeted therapies to ameliorate CVD has cast doubt on this HDL hypothesis [66,67]. A disconnection between the HDL cholesterol levels and the HDL atheroprotective functions may explain the findings obtained with therapies targeting HDL cholesterol.

**Figure 1:**
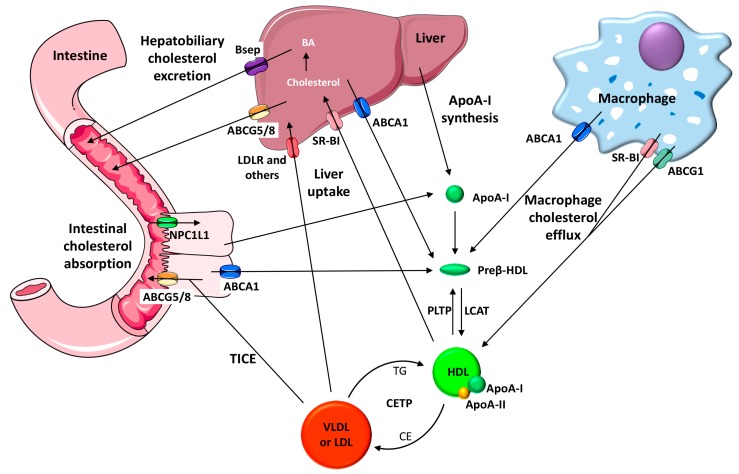
A schematic diagram of macrophage-to-feces reverses cholesterol transport pathways. ApoA-I is synthesized by the liver and small intestine, and it acquires phospholipids to become partially lipidated preβ-high-density lipoprotein (HDL) particles at its nascent stage. Preβ-HDL particles acquire free cholesterol from macrophages via the adenosine triphosphate-binding cassette (ABC) A1 transporter. The free cholesterol is converted into cholesteryl ester within the HDL particle by the action of Lecithin: cholesterol acyltransferase (LCAT), thereby resulting in mature HDL. ApoA-I is the major HDL protein and activates LCAT, whereas apoA-II, the second HDL protein, displaces apoA-I from the HDL particles. Both the macrophage scavenger receptor class B type I (SR-BI) and ABCG1 facilitate the cholesterol efflux process from macrophages to mature HDL. The phospholipid transfer protein (PLTP) promotes the transfer of phospholipids and free cholesterol from triglyceride-rich lipoproteins into HDL, producing a remodeling process where preβ-HDL particles can be generated. HDL cholesterol esters can be transferred to very low density lipoprotein (VLDL) or low-density lipoprotein (LDL) by the cholesteryl ester transfer protein (CETP) and be returned to the liver through the low-density lipoprotein receptor (LDLR) or other LDL and VLDL receptors. Another function of the liver is to take up HDL-associated cholesterol selectively via SR-BI. Cholesterol can be secreted into bile as unesterified cholesterol via the ABCG5/G8 heterodimer or used to synthesize bile acids (BA). The bile salt export pump (BSEP) is involved in the bile acid transport to bile. Niemann-Pick C1-like 1 (NPC1L1) is of crucial importance for absorbing macrophage-derived cholesterol in the small intestine. Cholesterol may also be excreted back to the lumen by the intestinal ABCG5/G8 heterodimer. The transintestinal cholesterol export (TICE) route promotes the flow of cholesterol from plasma to enterocytes and the intestinal lumen.

HDL is mainly synthesized in the liver and small intestine, and both may secrete lipid-free apolipoprotein (apo) A-I—the main HDL protein—into circulation [68]. The interaction produced between apoA-I and the transmembrane ATP-binding cassette (ABC) A1 induces cholesterol translocation to the cell membrane [68]. As a result, apoA-I is rapidly lipidized and converted into the nascent HDL (termed preβ-HDL). Cholesterol in these nascent discoidal particles is then esterified by Lecithin: cholesterol acyltransferase (LCAT). Cholesterol can then be taken up selectively from HDL after binding to the scavenger receptor class B type I (SR-BI) in the liver, captured by liver or kidney together with the whole HDL particle, or transferred by cholesteryl ester transfer protein (CETP) to very low density lipoprotein (VLDL) and LDL which may be later cleared by the liver via receptor-dependent pathways [68]. From there, it will be partly transformed into bile acids and removed together with cholesterol through the biliary pathway. Cholesterol and bile acids may then be finally excreted from the body in the feces. In addition to the main hepatobiliary pathway of cholesterol elimination, an alternative nonbiliary transintestinal route for cholesterol elimination has also been reported, termed the transintestinal cholesterol excretion (TICE) route. The TICE facilitates the transfer of cholesterol from the circulating plasma directly into the intestinal lumen through enterocytes [69]. All these multistep pathways have been collectively termed RCT (see Figure 1 for details). Whereas cholesterol efflux to HDL occurs in all tissues, the fraction that originates from the macrophage foam cells located in the arterial wall is considered the most critical RCT component directly related to atherosclerosis [70]. At least three cholesterol transporters are involved in the HDL-mediated macrophage cholesterol efflux: ABCA1, ABCG1 and SR-B1. ABCA1 promotes cholesterol transport to the lipid-free apoA-I, preβ-HDL and small HDL, whereas ABCG1 and SR-BI facilitate the efflux to mature α-migrating HDL [71]. It should also be noted that cholesterol efflux assays only quantify the first step of the atheroprotective RCT pathway without addressing the efficiency of the remaining RCT steps or other HDL atheroprotective properties, such as their antioxidant, anti-inflammatory and antithrombotic potential. An assay that evaluates the transfer of radiolabeled cholesterol from macrophages to feces has been widely applied to mice to determine the macrophage RCT rate of the entire pathway (Figure 1) [71]. This RCT multistep pathway, which is initiated by macrophages, is also susceptible to modulation in the liver and small intestine [69]. Studies investigating genetically engineered mice and mice treated with different RCT-enhancing therapies indicate that this major HDL antiatherogenic function is an important predictor of atherosclerosis susceptibility [72].

## 4. The Physiological Interaction between TMAO and HDL in the Context of Cardiometabolic Diseases

### 4.1. The Role of the Gut Microbiota in Lipid Metabolism and the Pathogenesis of CVD

Studies of gut microbiota composition and obesity have shown a direct association between Firmicutes phyla and obesity in both mice [73] and humans [74]. Dysbiosis has also been observed in other pathologies such as type 2 diabetes, where affected women had an increased *Clostridiales* colonisation at the expense of *Roseburia* [75]. A metagenomic study also showed differences in the gut metagenome of children with type 1 diabetes versus matched controls suggesting that environmental factors may interact with the genetic susceptibility to autoimmune diabetes [76].

The role of gut microbiota in lipid metabolism and in the pathogenesis of CVD has been widely investigated. A study performed on symptomatic atherosclerotic patients revealed a greater abundance of *Collinsella* than the control group, which showed enrichment in *Bacteroides, Eubacterium* and *Roseburia*—these three are all genera involved in anti-inflammatory and antioxidant processes [77]. Richness in different taxonomies of gut microbiota correlated negatively with body mass index and plasma triglycerides and positively with plasma HDL cholesterol. Hence, microbiota can explain a substantial proportion of the variation in lipid profile, independently of age, sex, body mass index and genetics [13]. A comparison between germ-free versus conventionally raised mice showed increased VLDL triglyceride production rates and hepatic triglyceride levels in the conventionally raised group [78].

Beyond the effects of intestinal microbiota on triglycerides and HDL cholesterol levels, the suppression of the intestinal microbiota was correlated with enhanced macrophage-to-feces RCT [79]. This change was concomitant with an increased bile acid excretion. Since the absence of gut bacteria impairs the secondary bile acid formation, the enhanced macrophage-to-feces RCT can be explained by the accumulation of hydrophilic tauro-β-muricholic acid, which cannot be absorbed in the colon [79]. However, plasma cholesterol levels and fecal neutral sterol excretion were not affected by the absence of intestinal microbiota [79]. In line with these findings, antibiotic treatment reversed the TMAO-dependent reduction of RCT [48].

Since the bile acid pool size and its composition seem to regulate the gut microbial community structure [80], the composition of the gut microbiota appears to be a key linking feature between TMAO production, and cholesterol and bile acid metabolism. Indeed, a lower microbial diversity and a greater enrichment of *Firmicutes* (relative to *Bacteroidetes*) were detected among healthy young men who exhibited a greater postprandial increase in circulating TMAO after consuming eggs and beef [34].

Several research groups have attempted to provide a mechanistic basis for TMAO-mediated atherosclerosis, where the variation in the gut microbiota could be a direct contributor to the pathogenesis and progression of the disease. Transferring choline diet-induced TMAO production via fecal transplantation in apoE-deficient (−/−) mice resulted in an increased atherosclerosis risk [81]. In the same line, a recent study with apoE−/− mice showed that the development of atherosclerosis by microbiota was dietary dependent [64]. Altogether, these findings suggest that elevated concentrations of circulating TMAO may arise from a dysbiotic microbiota, which in turn could be the cause underlying the pathogenesis of disease and its progression. Alternatively, circulating TMAO could reflect the differences in gut microbiota composition that occurs during disease process [82].

### 4.2. The Role of FMO3/TMAO and Nuclear Receptors on Enterohepatic Cholesterol and Bile Acid Metabolism

**Figure 2:**
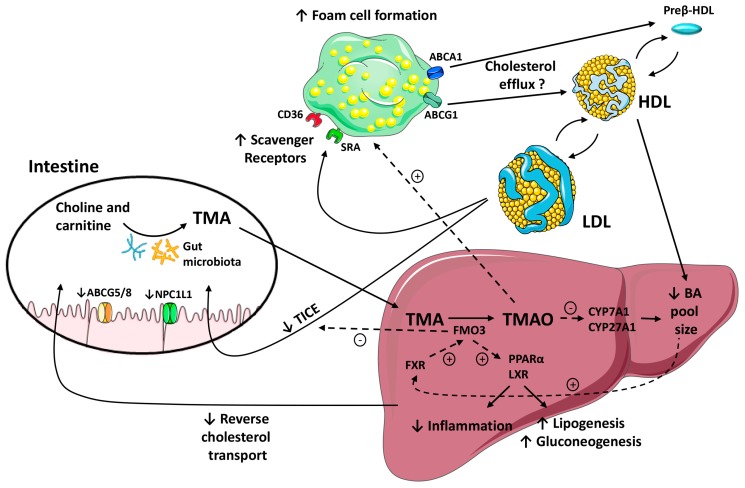
A schematic representation of pathways linking the gut microbiota, the formation of trimethylamine-*N*-oxide (TMAO) by flavin monooxygenase (FMO) 3 and the regulation of enterohepatic bile acid (BA) and cholesterol metabolism and macrophage reverse cholesterol transport (RCT) pathways. Black arrows indicate movement of TMA/TMAO and cholesterol through the body. Blunt-end arrows indicate activation (+) or inhibition (-) of specified receptors and transporters or pathways. Gut microbiota metabolism of choline and l-carnitine results in the formation of trimethylamine (TMA). In the liver, FMO3 converts TMA into TMAO. The potential effects of TMAO on the proatherogenic pathways include the promotion of foam cell formation by increasing macrophage scavenger receptors (↑) and the downregulation of the main liver bile acid (BA) synthetic enzymes, cyp7a1 and cyp27a1. Downregulation of these rate-limiting enzymes reduces intracellular levels of BA (↓). The BA pool size could impact the farnesoid X receptor (FXR)-mediated regulation of FMO3. In turn, this enzyme may regulate the liver X receptor (LXR) and peroxisome proliferator-activated receptor (PPAR) α signaling pathways, reducing liver inflammation (↓) and promoting hepatic lipogenesis and gluconeogenesis (↓). FMO3 impairs the cholesterol flux into the nonbiliary transintestinal cholesterol export (TICE) pathway (↓). TMAO also reduces Niemann-Pick C1-Like 1 (NPC1L1) and adenosine triphosphate-binding cassette (ABC) G5/G8 expression (↓), and inhibits intestinal cholesterol absorption. Collectively, the effects of TMAO/FMO3 on BA homeostasis and TICE impair the macrophage-to-feces RCT (↓).

Previous studies have demonstrated that the way TMAO impacts on atherosclerosis is closely connected to changes in bile acid metabolism [83]. TMAO reduced bile acid synthesis and liver bile acid transporters, effectively decreasing the bile acid pool [48]. The farnesoid X receptor (FXR) is a member of the nuclear receptor superfamily that acts as a sensor of intracellular bile acid levels within the liver and intestine [84]. Activation of FXR has been shown to cause significant changes in bile acid homeostasis by altering the transcription of genes responsible for liver bile acid synthesis and intestinal uptake [85]. Indeed, FXR downregulated the hepatic cytochrome P450 7A1 (CYP7A1, cholesterol 7 α-hydroxylase)—the rate-limiting enzyme in bile acid synthesis—through a fibroblast growth factor 15/19-dependent mechanism [86]. Interestingly, FMO3 expression in the liver was upregulated by dietary bile acids through an FXR-mediated pathway (Figure 2) [22]. In vivo studies have shown that a dietary TMAO supplementation inhibited bile acid synthesis by downregulating *cyp7a1* and *cyp27a1* (Figure 2) [48]. Furthermore, mice supplemented with TMAO had a significantly smaller total bile acid pool size concomitant with a reduced expression of the multiple liver bile acid transporters organic-anion-transporting polypeptide type 1 and 2, the multidrug resistance protein 2 and the sodium-taurocholate cotransporting polypeptide compared to control, chow-fed mice [48]. Dietary TMAO also reduced the mRNA expression of *Niemann-Pick C1-Like 1* and *abcg5/g8* and inhibited intestinal cholesterol absorption [48]. Since impaired bile acid synthesis and secretion are linked to an increased risk of CVD, the direct and indirect actions of TMAO on bile acid synthesis and excretion may represent a potential mechanism by which TMAO exerts its proatherogenic effect [48].

Recently, a gut microbial-driven pathway that balances the amount of cholesterol entering the biliary and nonbiliary pathways was identified. This study demonstrated that FOM3 inhibition diverted cholesterol into the nonbiliary TICE pathway, which resulted in the reorganization of the total body cholesterol balance [59]. The TICE is responsible for 30% of the total cholesterol loss through the feces in mice [87] and pharmacological liver X receptor (LXR) activation increased this amount up to 60% [88]. FMO3 knockdown mice presented an increased basal and LXR agonist-stimulated rate of the nonbiliary TICE pathway, which are findings that further support FMO3 as a negative regulator of the TICE (Figure 2). Moreover, knocking down FMO3 in mice strikingly reduced the expression of LXR target genes involved in the de novo lipogenesis and blunted hepatic steatosis, in contrast, it exacerbated hepatic endoplasmic reticulum stress and inflammation [59]. Overall, FMO3 activity and the TMA/FMO3/TMAO pathway seem to be major determinants for both LXR and FXR regulation as well as for the downstream liver inflammatory response (Figure 2). Therefore, all of these pathways have broad implications in sterol balance and inflammatory processes, suggesting that FMO3 has regulatory functions distinct from its enzymatic activity, being uniquely positioned among the FMO family of enzymes in impacting human disease [22,48].

Dietary TMAO has been found to normalize plasma levels of circulating TMAO in FMO3 antisense oligonucleotide (ASO)-treated mice, but neither cholesterol balance nor gene expression in the liver [48]. This indicates that the ability of FMO3 inhibitors to alter cholesterol balance, inflammation and endoplasmic reticulum stress is likely to involve several molecular mechanisms, including both the gut microbial and the gut microbe-independent mechanisms [59].

Knocking down liver FMO3 in LDL receptor-deficient mice reduced the hepatic and plasma lipid levels, bile acid pool size, liver triglyceride secretion, ketone bodies and glucose and insulin levels, and prevented atherosclerosis [89]. Global microarray expression analyses of these mice revealed that knocking down liver FMO3 downregulated 136 peroxisome proliferator-activated receptor (PPARα) target genes, possibly due to reduced hepatic concentrations of PPAR ligands, which included palmitoleate, α- or γ-linolenate, oleate and eicosapentaenoic acid [89,90]. PPARα is activated during fasting and may promote fatty acid oxidation, ketogenesis, gluconeogenesis and bile acid synthesis through transactivation of its target genes [90], thus explaining many of the FMO3-mediated effects. It is thus becoming increasingly important to identify endogenous substrates—these can include epinephrine, norepinephrine, phenethylamine, trimethylamine and tyramine or new potential substrates; and products of FMO3 that have the potential to differentially impact on the diverse phenotypes observed when manipulating this enzyme.

As stated above, elevated systemic levels of TMAO have been associated with type 2 diabetes [91]. Moreover, FMO3 was increased in obese-/insulin-resistant human subjects. Interestingly, knocking down FMO3 prevented high-fat-diet-induced obesity in mice by stimulating the beiging of white adipose tissue [91]. Furthermore, knocking down FMO3 in liver-specific insulin receptor knockout mice prevented hyperlipidemia and atherosclerosis susceptibility concomitant with an increase in LDL receptors [92]. These findings concurred with the results of a previous report demonstrating the deleterious effects of FMO3 expression in glucose tolerance in liver-specific insulin receptor knockout mice [92]. Additionally, another recent study demonstrated that maternal hypercholesterolemia exacerbates the development of atherosclerosis with a positive association of aortic lesion size with both TMAO levels and increased FMO3 mRNA expression [93]. Overall, these data strongly indicate a role for FMO3 in modulating lipid and glucose homeostasis in vivo in a dose-dependent manner and, in some cases, independently of TMA/TMAO formation.

### 4.3. TMAO/FMO3 and HDL Atheroprotective Functions

As stated above, several studies have demonstrated that TMAO can promote macrophage cholesterol accumulation in a microbiota-dependent manner by increasing cell surface expression of two scavenger receptors [14]. However, TMAO treatment in apoE−/− mice failed to impair the macrophage cholesterol efflux, at least in part due to upregulation of ABCA1 and ABCG1 in peritoneal mouse macrophages and increased ABCA1-dependent cholesterol efflux to ApoA-I [48]. Nonetheless, another study found downregulation of the *abca1* gene in J774.A1 murine macrophages treated with TMAO for 24 h [94] but this finding was not reproduced in another similar study [63].

Beyond the potential effect of TMAO on macrophage cholesterol efflux, mice fed with a choline- or l-carnitine-enriched diet showed an impaired macrophage-to-feces RCT in vivo [48]. This process was reverted when a broad spectrum of antibiotics was administered orally, suggesting that TMAO was the main responsible factor of altering the entire macrophage RCT pathway [48]. Consistent with this hypothesis, mice directly fed with a TMAO-containing diet al.so showed a reduced rate of macrophage-specific RCT [48]. As discussed earlier, TMAO-mediated effects on the liver bile acid synthetic pathway could be affecting the recovery of macrophage-derived cholesterol in feces (Figure 2).

A liver microarray analysis was conducted in two independent mouse models with an enhanced TICE to identify potential regulators of macrophage RCT. Less than 100 differentially expressed genes were detected within each array data set [59]. From these, the only gene that was downregulated in both models was FMO3. Consistently, when FMO3 was suppressed, the nonbiliary macrophage-to-feces RCT was enhanced [59]. However, it was not possible to determine which step of the RCT was altered because knocking down FMO3 substantially impacted the whole-body cholesterol balance [59]. Further experiments should be performed to clarify how the FMO3/TMA/TMAO pathway affects the macrophage RCT.

## 5. Concluding Remarks

There is significant evidence supporting the hypothesis that gut microbiota-derived production of TMAO increases the risk of atherosclerotic CVD. The concentration of circulating TMAO is determined by several main factors, including the dietary habits, gut microbiota, FMO3 activity and kidney function. Several important published works in TMAO research have used omics-techniques, mainly focusing on its involvement in CVD (see Table 1 for references of clinical studies). These technologies have been fundamental in the identification of TMAO origin and in the characterization of the unique properties of FMO3, the enzyme that ultimately synthesized TMAO. TMAO can promote atherogenesis via multiple signaling pathways. Dietary administration of choline, l-carnitine or TMAO in animals with an intact gut microbiota enhances the macrophage scavenger receptors and promotes foam cell formation. However, the diets enriched in TMAO precursors have not always been found to be proatherogenic in experimental studies. This dietary administration impairs the macrophage-to-feces RCT pathway. Furthermore, TMAO impairs the FXR-target genes *cyp7a1* and *cyp27a1*, which affect liver cholesterol, bile acid production and biliary excretion. FMO3 may also promote atherosclerosis by enhancing hepatic lipogenesis and gluconeogenesis as well as by impairing the TICE. At least a part of the FMO3-mediated effects on lipid metabolism seems to be independent of TMA/TMAO formation, suggesting multiple distinct mechanistic links between the TMAO-producing diet, FMO3 and atherogenesis (see Figure 2 for a graphical summary). Overall, and based on the highly variable plasma TMAO levels observed in human studies, it is reasonable to hypothesize that TMAO levels are affected by the intrinsic genetic factors of the host and that FMO3 activity may influence pathological outcomes via routes independent of TMAO. However, diets containing TMAO precursors provide important healthy nutrients and, thus, proposing restriction of these foods does not seem a suitable strategy. Further human studies that investigate the effects of lowering circulating TMAO on CVD are needed. Based on the promising available data regarding gut microbiota to date, their selective manipulation could be used as a therapeutic approach in future therapies to prevent atherosclerotic CVD.

## Figures and Tables

**Table 1:** Examples of studies using one or more multi-omics technologies investigating the pathophysiological role of TMAO.

Methods and Species	Study Findings	References
Metabolomics in humans	TMAO, carnitine, choline and phosphatidylcholine plasma levels correlated with CVD	[14,48]
GWAS in mice	The *Slc30a7* gene was related to TMAO metabolism	[44]
GWAS in humans	*SLC30A7* locus did not reach the genome-wide significance	[45]
Epigenome-wide study of DNA methylation in humans	No evidence of significant relationship between methylation markers and circulating TMAO levels	[47]
A multi-omic association study of TMAO in humans	Diet- and disease-associated metabolites were significantly associated with TMAO Proteins linked to CVD and kidney disease were correlated with TMAO	[30]
Metagenome analysis in humans	Gene-targeted sequencing allowed the quantification and characterization TMA-producing species	[37]
Metagenome analysis in humans	Gut microbiome may explain part of the population variation in plasma blood lipids	[13]
Global microarray expression analyses in mice	Knocking down FMO3 downregulated 136 PPARα target genes	[89]
Global microarray expression analyses in mice	FMO3 expression was the unique downregulated gene in mouse models of enhanced TICE	[59]

## Abbreviations

ABC	ATP-binding cassette
ASO	Antisense oligonucleotide
CD36	Cluster of differentiation 36
CETP	Cholesteryl ester transfer protein
CKD	Chronic kidney disease
CVD	Cardiovascular disease
FMO	Flavin monooxygenase
FXR	Farnesoid X receptor
GWAS	Genome-wide association studies
HDL	High-density lipoprotein
LCAT	Lecithin cholesterol acyltransferase
LDL	Low-density lipoprotein
LXR	Liver X receptor
MACE	Major adverse cardiovascular events
mRNA	Messenger ribonucleic acid
Npc1L1	Niemann-Pick C1-Like 1
OCT2	Organic cation transporter 2
PPAR	Peroxisome proliferator-activated receptor
SR-BI	Scavenger receptor class B type I
TICE	Transintestinal cholesterol excretion
TMA	Trimethylamine
TMAO	Trimethylamine-*N*-oxide
VLDL	Very low-density lipoprotein
γBB	γ-Butyrobetaine
